# Integrating substance use disorder services into public primary healthcare: a case study from Lebanon

**DOI:** 10.3389/fpubh.2026.1824996

**Published:** 2026-04-16

**Authors:** Tatyana Sleiman, Najwa Nassif, Dala Fakhreddine, Ramzi Haddad

**Affiliations:** 1 SKOUN, Beirut, Lebanon; 2 Department of Psychiatry, Lebanese University, Beirut, Lebanon

**Keywords:** integrated care, Lebanon, primary health, public health, substance use disorder

## Abstract

In Lebanon, economic collapse, political instability, and a strained public health system have left people who use drugs (PWUD) with minimal access to care, while stigma and punitive drug laws further prevent their willingness to seek help. In this fragile context, integrating mental health and substance use care within primary healthcare centers (PHCCs) represents a promising approach to addressing unmet needs by leveraging existing infrastructure to deliver accessible, coordinated, and stigma-reducing care. To address this gap, Skoun and Médecins du Monde (MdM) introduced substance use and mental health services into PHCCs. Skoun was responsible for integrating substance use care and delivering treatment services. The intervention employed a three-pronged strategy: (1) training healthcare providers at PHCCs, (2) establishing institutional partnerships and co-management with PHCCs, and (3) developing locally contextualized clinical and operational guidelines. Two pilot community mental health and substance use centers (CMH-SUCs) were established within existing PHCCs. The early outcomes are promising, as reflected in the number of people who benefited from Skoun’s services and in the improved capacity of PHCC providers to understand harm reduction and manage substance use within primary care. The pilot project demonstrated that integrating substance use disorder (SUD) care within PHCCs is feasible and effective, even in fragile settings. It enhanced access to services and strengthened coordination between governmental and non-governmental sectors. However, persistent barriers, such as stigma, restrictive drug laws, workforce instability, and fragmented funding, underscore the need for systemic reform. Embedding SUD services within primary care, supported by national policy change and sustainable financing, may offer a pathway toward equitable, rights-based, and cost-efficient drug policy reform in Lebanon and similar contexts.

## Introduction

### Why integration matters in fragile health systems

Integrated treatment models, in which mental health and substance use disorder (SUD) services are delivered within a coordinated or unified framework, have been shown to improve patient outcomes compared to parallel or sequential care. Integrated care reduces treatment fragmentation, enhances engagement, and improves both substance use and mental health outcomes (1). This approach promotes holistic, person-centered care by coordinating services across mental health, substance use, housing, and social support systems, thereby creating more seamless and sustained recovery pathways (2). Additionally, integrated care can reduce costs and increase long-term efficiency, strengthening the economic rationale for integration (3). Extending this integration into primary healthcare offers further advantages. Primary care is often the first and most consistent point of contact for patients and is uniquely positioned to provide early identification, brief interventions, and ongoing management of SUD and co-occurring mental health conditions. Embedding behavioral health and SUD treatment into primary care improves access, particularly for underserved populations, reduces stigma, and supports more holistic care (4, 5). In addition, integrating services into primary care leverages existing infrastructure, workforce, and patient relationships, enabling scalable and sustainable approaches to address the intertwined epidemics of substance use and mental illness (6). In fragile and conflict-affected settings, integration of mental health services into primary healthcare has been widely promoted as a pragmatic response to the severe shortage of specialist providers and the disruption in health systems. Most efforts to achieve this integration have involved implementing of the World Health Organization (WHO) Mental Health Gap Action Programme (mhGAP). Evidence from fragile states in Africa, including Somalia and Sudan, suggests that mhGAP-based training and supervision can improve providers’ knowledge, confidence, and basic service delivery despite insecurity and limited infrastructure (7, 8). In the Gaza Strip, a stepped-care integration model showed successful screening and implementation following mhGAP-aligned training and supervision (9). In Lebanon, the integration of mental health into primary healthcare was operationalized through mhGAP-aligned training, supervision, and the development of referral pathways within primary healthcare centers (PHCCs) (10). However, across these settings, the integration efforts are far more advanced for common and severe mental disorders than for SUDs.

### Substance use in Lebanon

Lebanon lacks nationally representative epidemiological data on substance use, which remains a significant limitation for effective policymaking and service planning. An international study, including Lebanon and led by the World Health Organization, found a cumulative incidence of usage of 53.3% for alcohol, 67.4% for tobacco, 4.6% for cannabis, and 0.7% for cocaine (11). Treatment-seeking trends and data from few centers in Lebanon that offer treatment and support for people who use drugs (PWUD) suggest a consistent rise in both the use and misuse of licit and illicit substances since the early 2000s (12). Data are collected from treatment centers that are almost exclusively located in Beirut and Mount Lebanon (13); therefore, there is a lack of data on the drug use situation in Lebanon’s North, South, and Bekaa regions.

### A neglected public health crisis

Substance use in Lebanon must be understood within the context of its turbulent history. The Lebanese Civil War (1975–1990) not only devastated the country’s infrastructure but also contributed to the rise of drug trafficking and consumption. During the conflict, militias and foreign actors facilitated opium and hashish production in the Bekaa Valley, embedding drug economies within Lebanon’s war economy (14). In the post-war years, weak governance and underfunded social services failed to address the psychosocial traumas that fueled substance use, leaving a gap in mental health and addiction support services. Moreover, substance use in Lebanon is a social justice issue, deeply shaped by stigma, criminalization, and neglect (13). Lebanese Law No. 673, passed in 1998, criminalizes drug use and possession, imposing imprisonment of up to 3 years. However, the law allows for an alternative to incarceration through referral to treatment. In practice, this provision is rarely applied due to a lack of publicly funded treatment options. Courts often default to incarceration, and treatment referrals rely almost exclusively on non-governmental organizations (NGOs) (15). This gap between policy and practice further marginalizes PWUD, restricting their access to healthcare, legal assistance, and social support systems (16). The structural challenges and legal ambiguity are compounded by the absence of a comprehensive state-led strategy to address substance use. Although Lebanon launched the National Substance Use Strategy (17), its implementation has been minimal due to political instability and insufficient funding. The 2019–2020 economic crisis, compounded by the COVID-19 pandemic and the Beirut Port Explosion, further strained state and healthcare institutions, deepening existing challenges. Public health facilities collapsed under financial pressures, leading to the further disintegration of an already limited mental health infrastructure (18). The crisis unveiled how Lebanon’s healthcare system, particularly in its public form, had no coherent mechanism for addressing substance use and mental health concurrently.

The primary goal of the initiative was to embed a sustainable, community-based model for mental health and substance use services within Lebanon’s fragile yet essential public health infrastructure. This model aimed to reduce the isolation and stigmatization of PWUD, increase access to evidence-based treatment, and standardize care pathways across PHCCs.

## Methods

### Context

Lebanon’s healthcare system is marked by a stark divide between the public and private sectors. Although the country has a relatively dense network of health facilities, the system is highly privatized and heavily reliant on out-of-pocket expenditures. According to the World Bank, private spending, including out-of-pocket payments, private health insurance, and contributions from local NGOs, accounts for 50.7% of total health expenditure (19). The Ministry of Public Health plays a regulatory and subsidizing role rather than providing comprehensive services. PHCCs, a network of over 200 centers coordinated by the MoPH, serve as the backbone of Lebanon’s public health response. These facilities provide basic preventive and curative services, including maternal health, vaccinations, and non-communicable disease management. However, up until 2018, PHCCs were underutilized by Lebanese citizens, who tended to prefer private clinics despite higher costs (20). During the crisis period beginning in 2019, the role of PHCCs shifted dramatically. As the Lebanese pound lost over 90% of its value and private healthcare became prohibitively expensive, Lebanese citizens increasingly turned to PHCCs for care. According to the United Nations High Commissioner for Refugees, by 2022, the number of Lebanese citizens accessing PHCCs had tripled (21). Until 2019, substance use services were practically non-existent in public health facilities, except for a few governmental hospitals that offered inpatient detox treatment through their psychiatric wards. Treatment for SUD was limited to a few psychiatric hospitals and private rehabilitation centers, most of which were unaffordable for the majority of support seekers. As a result, PWUD relied heavily on NGOs, which operated independently of national health policy frameworks. There were no integrated SUD treatment services within PHCCs (15).

### Process

In response to the ongoing economic collapse and its impact on communities and public health infrastructure, the rationale for integrating SUD treatment into primary healthcare systems was rooted in both efficiency and equity. Furthermore, Lebanon’s high concentration of refugees and marginalized groups requires an adaptable and decentralized approach to substance use care. Therefore, amid this crisis, there was an opportunity, perhaps unprecedented, to reimagine health services through a model in which public clinics could offer a cost-effective, socially inclusive, and health-promoting approach aligned with international best practices.

#### Skoun, Lebanese addictions center

Skoun is a Beirut-based NGO founded in 2003, operating both as a central outpatient treatment center and the leading organization integrating substance use and harm reduction community-based services into primary healthcare centers across multiple regions in Lebanon. Although its main clinical site is in Beirut, Skoun has historically extended its reach through outreach programs, partnerships with local organizations, and service delivery in underserved areas. Annually, Skoun provides services to over 4,000 beneficiaries through its prevention, treatment, and harm reduction programs. Skoun’s presence in marginalized communities, including refugees and low-income neighborhoods in Beirut and other urban centers, has been crucial (22). Moreover, Skoun also plays a pivotal advocacy role, promoting the decriminalization of drug use and the full implementation of Lebanon’s legal mandate prioritizing treatment over incarceration (23).

#### The community mental health and substance use center (CMH-SUC) model: origins, partnerships, and structure

Skoun has worked to foster partnerships with healthcare institutions and municipalities, laying the groundwork for what could become a model of community-based, integrated care. However, the limitations of Lebanon’s healthcare infrastructure, political instability, and legal constraints have hindered the scalability of these efforts. The initiative to integrate substance use response into Lebanon’s public primary healthcare system emerged from an urgent recognition of both structural gaps and growing health needs. Spearheaded by Skoun in partnership with Médecins du Monde (MdM) and the National Mental Health Program (NMHP), and supported by the Agence Française de Développement (AFD), the integration effort formally began in 2018 and gained significant momentum during the 2020 crisis, which catalyzed a shift in public health priorities.

The integration into PHCCs did not aim to shift patients from Skoun to public facilities but rather to expand access both geographically and structurally. Prior to the pilot, most services were concentrated in Beirut, creating significant barriers for populations in other regions of the country. The PHCC integration allowed new populations to access services locally, while also reducing the burden on centralized NGO services. The pilot focused on the areas of Tripoli in northern Lebanon and Douris–Baalbek in the Bekaa region. These areas were selected due to the unavailability of similar services and the high level of need among the population, driven by poverty, lack of access to education and employment, and the high availability of drugs, as both regions are hubs for the drug trade and smuggling routes. Moreover, both areas represent bordering regions that have been particularly affected by ongoing wars and security events. The strategy was multidimensional, grounded in Skoun and MdM’s field expertise, and aligned with national policy. It involved three core strategies:

##### Training and sensitization of healthcare providers

A cornerstone of the integration was capacity building for PHCC staff. Education and shared care models among non-addiction specialists have been shown to improve attitudes toward working with patients with SUDs (24). The process began with an evaluation to assess baseline knowledge and capacities. Based on the evaluation results and group discussions with PHCC and CMH-SUC staff, training topics and content were developed and adapted by Skoun’s clinical team. Training modules were designed to enhance the understanding of harm reduction, SUDs, and mental health while challenging stigma among providers. The sessions focused on clinical protocols, motivational interviewing, psychosocial assessment, and ethical engagement with PWUD.

Furthermore, general practitioners, nurses, and social workers were trained in the initial identification of people with SUD and any associated comorbidities, using a structured interview and risk assessment developed collaboratively by the clinical teams of Skoun and MdM.

Following training, Skoun’s clinical team conducted regular supervision sessions to support and follow up on the implementation of the newly acquired knowledge and practices.

##### Institutional partnerships with public healthcare centers

The initiative relied on strong partnerships with selected PHCCs. Skoun and MdM, in collaboration with the NMHP, worked with local health authorities and municipal leadership to identify PHCCs that were both willing and structurally capable of hosting CMH-SUCs. These collaborations were formalized through memoranda of understanding and included on-site support and shared management responsibilities.

##### Development of contextualized guidelines

**Figure 1:**
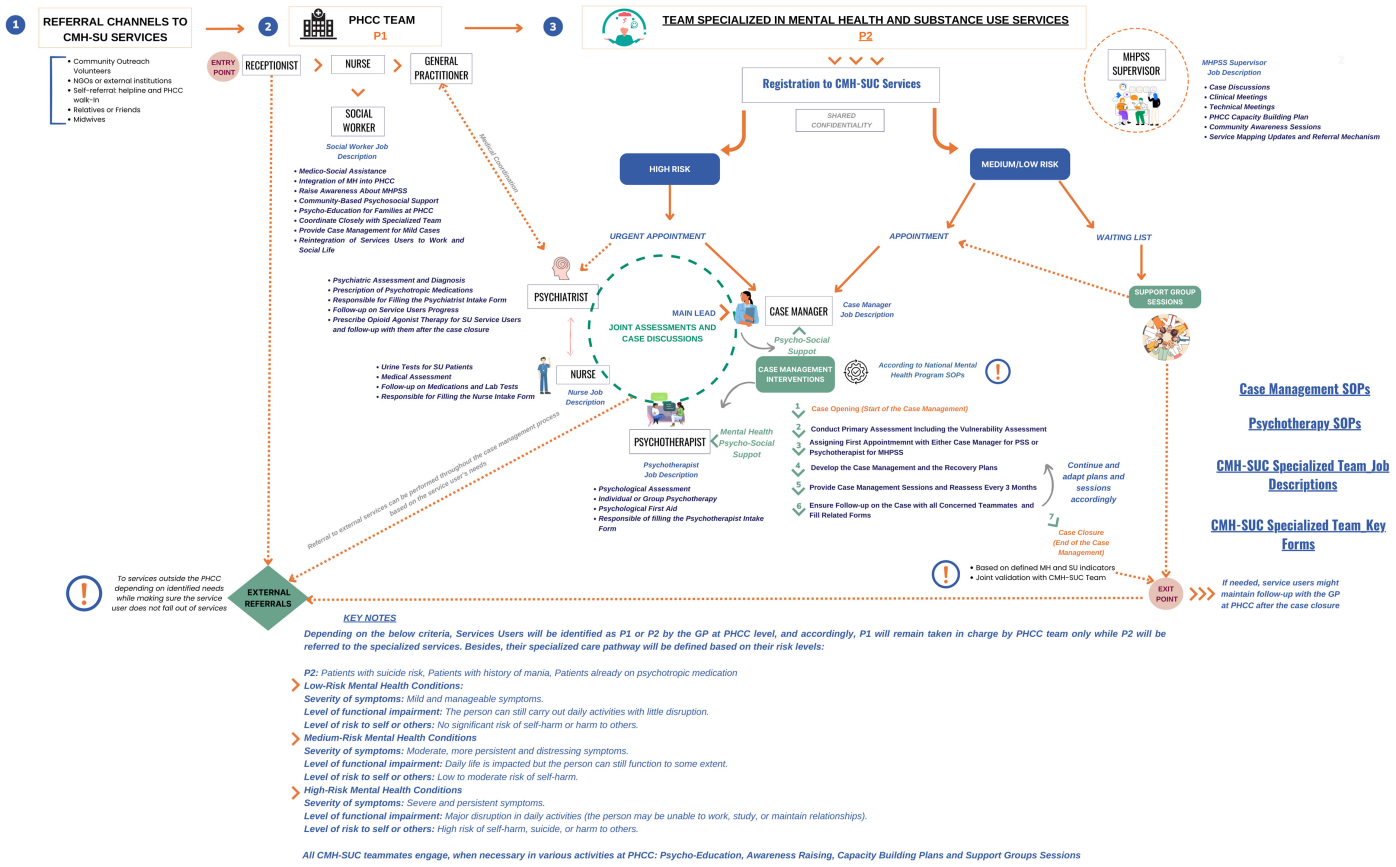
Integrated community mental health and substance use services: a detailed workflow toolkit.

Nationally tailored Standard Operating Procedures and clinical workflows were developed and adopted. These guidelines aligned with WHO recommendations for integrated care and were designed to reflect local realities. A detailed interdisciplinary workflow was created (Figure 1), enabling joint case discussions, data sharing, and referral mechanisms both within and beyond the PHCCs.

#### Comprehensive service care delivery

In total, two fully integrated pilot CMH-SUCs were launched in Tripoli (Al Rahma PHCC) and Baalbek–Douris (Mother and Child PHCC). These locations were strategically selected based on population vulnerability (including refugees and economically disadvantaged Lebanese individuals), readiness of PHCC infrastructure, and existing community ties with implementing NGOs.

The CMH-SUCs delivered a comprehensive, multidisciplinary service package, reflecting both harm reduction and mental healthcare models.

##### Counseling and psychosocial support

Each center offered case management, individual psychotherapy, family interventions, and psychoeducation. These services addressed the psychological dimensions of substance use, with ongoing assessments to ensure responsiveness to client needs.

##### Opioid agonist treatment (OAT)

Many integrated models of care for the treatment of opioid use disorder can be implemented according to available resources (25). Integrating buprenorphine treatment with primary care has been associated with greater retention in treatment (26). Accordingly, opioid agonist treatment (OAT) using buprenorphine, the only OAT medication available in Lebanon, was integrated into PHCC services. Psychiatrists embedded within the CMH-SUCs prescribed and monitored these treatments.

##### Referral systems and continuum of care

A robust referral network was established to connect services across multiple levels of care. This included the following:

- Referrals from PHCCs to CMH-SUCs for specialized substance use support.
- Referrals from CMH-SUCs to PHCCs for primary medical care, diagnostics, and pharmacological management.
- Linkages with external services, including hospitals, social service agencies, and legal aid.

This bidirectional referral system ensured continuity and avoided fragmentation, making the care more person-centered and holistic.

##### Community outreach and peer-led harm reduction

Beyond clinical settings, Skoun and its partners extended harm reduction services through community-based outreach. Outreach workers, including trained peers, conducted street and community visits to reach populations not in contact with formal care. These teams provided voluntary counseling and testing (VCT) for HIV, HBV, and HCV, shared information on overdose prevention, and delivered awareness sessions on the risks associated with unsafe drug use. Educational sessions also emphasized safer injection practices, available support services, and steps to prevent and respond to overdose situations.

The integration model prioritized cost efficiency by leveraging existing infrastructure and minimizing duplication. Instead of creating parallel drug treatment systems, the initiative built upon PHCCs’ reach and legitimacy in communities. This significantly reduced the per-patient cost of delivering care and expanded access for underserved populations, including low-income Lebanese individuals and Syrian refugees.

Moreover, integrating mental health and substance use care de-stigmatized treatment by normalizing it within public health services, making it more acceptable and accessible to the general population (27).

## Results

According to internal monitoring reports, during the first 2 years of operation:

- 270 individuals received treatment across the two centers.
- 6,305 SU-related consultations were offered, including psychotherapy, nursing interventions, case management, and psychiatric consultations.
- 16,039 individuals were reached through outreach and street-based interventions.
- HIV and HCV testing services were utilized by 903 individuals, many of whom had never previously accessed voluntary testing.

Furthermore, unpublished data from Skoun’s service delivery system provide critical insights into patterns not captured in surveys and highlight regional disparities. Specifically, Skoun’s internal data comparing types of substances used among beneficiaries in Beirut, Tripoli, and Baalbek show higher rates of methamphetamine use in Tripoli and Baalbek, likely reflecting increased availability linked to trafficking routes. In contrast, in Beirut and Mount Lebanon, alcohol and cocaine use are more prevalent, patterns often associated with urban environments and differing socio-economic profiles. These distinctions are consistent with both field observations and programmatic data.

### Barriers and challenges

Implementing and sustaining integrated services faces several challenges and barriers, similar across both regions, including persistent stigma among communities and some healthcare providers, legal ambiguities related to drug use criminalization, limited and unstable funding, and shortages of trained personnel.

Although these challenges were common to both pilot sites, their expression differed contextually. In Tripoli, stigma and community resistance were more pronounced during the early phases, requiring intensive community engagement. In Baalbek, logistical challenges, particularly related to resource availability and geographic accessibility, were more prominent. These contextual differences influenced the pace of implementation and service uptake.

### Outcomes

Despite these challenges, early indicators point to positive outcome trends among service users engaged in the integrated model, where a large percentage of PWUD accessing counseling and psychosocial services reported improvements. To measure improvement, Skoun has established a comprehensive monitoring system, beginning with an intake form for each new patient that provides baseline information on substance use levels. Following treatment initiation, follow-up assessments are conducted at 3, 6, and 12 months. These assessments track changes in patients’ substance use levels relative to their answers in the intake form and evaluate whether patients have achieved their specific treatment goals based on self-reported perceptions. The vast majority of patients (98% in Tripoli and 95.5% in Baalbek) reported improvements in their well-being during the treatment process.

The project also led to measurable improvements in awareness, attitudes, and competencies among healthcare providers in public facilities. A total of 120 doctors, nurses, and social workers from public PHCCs participated in structured capacity-building workshops developed by Skoun and MdM. Post-training evaluations demonstrated the following:

- 87% of participants reported an improved understanding of harm reduction.
- 72% stated they felt more confident managing substance use cases within their primary care practice.
- Providers’ willingness to refer or engage with PWUD increased notably.

## Discussion

The initiative, led by Skoun and MdM in partnership with NMHP-MoPH and supported by the AFD, yielded promising outcomes. These outcomes provide strong evidence of the potential scalability and sustainability of community-based mental health and substance use care models in fragile contexts such as Lebanon.

An important indicator of success has been the increased access to substance use services through PHCCs. Through the pilot CMH-SUCs established in Tripoli (Al Rahma PHCC) and Baalbek (Mother and Child PHCC), hundreds of individuals received services, information, and psychosocial support that were previously unavailable in the public sector. These figures are especially relevant because of the historical gap in harm reduction and SUD services available to vulnerable populations outside Beirut. Prior to this pilot, no similar services were available within these PHCCs or across the broader geographical areas they serve.

The availability of these services outside the capital and the advocacy accompanying this process also prompted the MoPH to expand the network of registered dispensaries for OAT treatment—from two dispensaries in Beirut at the beginning of the pilot to seven dispensaries nationwide by the time this case study was developed.

Furthermore, the initiative catalyzed a notable increase in referrals between PHCCs and CMH-SUCs, illustrating enhanced health system integration and growing trust among healthcare providers. This shift in referral patterns also reflects a broader change in perceptions among public healthcare workers, representing an essential step toward the gradual normalization of harm reduction approaches within Lebanon’s health system as an evidence-based and reliable health response to the needs of PWUD.

Another major achievement has been the strengthening of multisectoral collaboration between public institutions, local NGOs, and international donors. Skoun’s formal partnership with the MoPH, through the NMHP, has elevated the legitimacy of SUD treatment and harm reduction services as a public health priority. This collaboration has helped integrate substance use interventions into national policy discussions on the expansion of PHCC services, particularly within Lebanon’s broader mental health and non-communicable disease frameworks.

Support from the AFD and MdM was also instrumental in providing the technical guidance, training, and flexible funding needed to implement the pilot successfully. This multistakeholder model, combining technical expertise and grassroots implementation capacity, offers a promising blueprint for scaling up integrated care across the country.

At the time of writing this article, a new donor and international agency have adopted the model presented herein to integrate mental health and substance use care into two additional PHCCs. There are plans to formalize the use of validated screening tools within PHCCs to improve early detection, standardization, and monitoring of SUD and co-occurring mental health conditions. This expansion reflects growing institutional interest in the model’s efficacy and its relevance to addressing Lebanon’s complex health challenges. Meanwhile, the two original PHCCs in Tripoli and Baalbek continue to operate the integrated services at varying levels of capacity, depending on available resources. Although technical support from Skoun and the NMHP has been reduced, the services remain functional, an encouraging indicator of local ownership and the institutionalization of the model. Continued investment and support will be crucial to ensuring the sustainability and quality of these services as Lebanon navigates its ongoing socio-economic crisis.

A review of barriers to the integration of substance use treatment services into primary care identified numerous challenges for patients, providers, and programs/systems (28). In Lebanon, barriers exist across cultural, legal, and operational domains, each of which significantly impacts implementation and sustainability. Research shows that stigmatizing attitudes toward people with SUDs are common among healthcare professionals (29). These attitudes are associated with less involvement in addiction care (30). In Lebanon, stigma is still a major concern affecting people with mental health disorders (31). Substance use is still largely perceived through a moralistic or criminal lens rather than as a health issue. Efforts to sensitize and engage communities, particularly through outreach and dialogue with local influencers, have helped mitigate some of this resistance. However, deeply ingrained social norms remain a substantial challenge that requires ongoing education and advocacy.

Lebanon’s legal framework also presents significant regulatory obstacles to the full realization of integrated harm reduction and SUD treatment services within public healthcare. The law’s punitive foundation frames substance use primarily as a criminal offense rather than a public health concern, reinforces stigma, deters individuals from seeking help, and hinders state financial investment in support structures and mechanisms.

Financial barriers and inadequate or inflexible funding have been mentioned among the most commonly reported barriers to integrated treatments (32, 33). Lebanon’s national healthcare regulations do not formally mandate, nor institutionalize, SUD treatment or harm reduction services. Most interventions, such as OAT, HIV testing, or psychosocial support for PWUD, are not part of MoPH-funded service packages. Furthermore, another key barrier is ensuring service continuity amid shifting donor landscapes and funding uncertainties. The introduction of specialized services such as OAT, psychotherapy, and urine testing requires specialized training, equipment, and monitoring, all of which place additional burdens on health centers. Currently, Lebanese regulations allow OAT prescriptions to be issued only by psychiatrists. Nevertheless, there is increasing recognition, both within the MoPH and among stakeholders, of the need to expand the eligibility of prescribers. Future scale-up efforts may advocate for regulatory adaptations that allow trained general practitioners to prescribe and monitor OAT, which would significantly enhance access and sustainability.

Furthermore, staff turnover, often due to burnout or lack of funding, disrupts the continuity of care and reduces the institutional memory necessary to sustain newly integrated health service models.

The lack of an updated national drug strategy that is fully operationalized and aligned with international best practices also inhibits systemic reform. Although the National Substance Use Strategy (2016–2021) outlines clear priorities for reducing stigma, improving access to care, and integrating services, it has neither been meaningfully implemented nor updated yet due to political instability, resource constraints, and competing priorities.

## Conclusion

The integration of harm reduction and SUD services into Lebanon’s public primary healthcare system through the CMH-SUC pilot demonstrates that, even in fragile, resource-limited contexts, meaningful change is possible when public institutions, NGOs, and international partners collaborate under a shared vision. This initiative has expanded access for marginalized populations and created new pathways for coordinated, person-centered care. Early results indicate improved service uptake, stronger provider competencies, and better continuity of care for PWUD—outcomes that were previously out of reach within Lebanon’s fragmented system.

However, the experience also underscores persistent challenges. Deeply rooted stigma, restrictive legal frameworks, inconsistent funding, and the absence of a fully implemented national drug strategy remain significant barriers to scale-up and sustainability. Addressing these issues will require sustained advocacy, legal reform, secure financing mechanisms, and the institutionalization of harm reduction within national health policy frameworks.

Ultimately, the pilot offers a replicable model for integrating mental health and SUD services into primary care that is both cost-effective and socially inclusive, addressing the intertwined public health challenges of substance use and mental illness in Lebanon.

## Data Availability

The raw data supporting the conclusions of this article will be made available by the authors, without undue reservation.
